# Core Competencies for Gerontogeriatric Nursing: A Validation Study

**DOI:** 10.3390/geriatrics9030073

**Published:** 2024-05-31

**Authors:** Maria José Catalão, Helena Arco, Nuno Carrajola, Maria de Lurdes Almeida, Hugo Neves, João Tavares

**Affiliations:** 1 Department of Health Sciences and Technologies, School of Health, Portalegre Polytechnic University, Campus Politécnico 10, 7300-555 Portalegre, Portugal; helenarco@ipportalegre.pt (H.A.); nuno.carrajola@ipportalegre.pt (N.C.); 2 CARE—Research Center on Health and Social Sciences, Portalegre Polytechnic University, 7300-555 Portalegre, Portugal; 3 Department of Education and Psychology, University of Aveiro, 3810-198 Aveiro, Portugal; 4 ICBAS, University of Porto, 4050-313 Porto, Portugal; 5 Health Sciences Research Unit: Nursing—UICISA, Nursing School of Coimbra, 3046-851 Coimbra, Portugal; mlurdes@esenfc.pt (M.d.L.A.); hugoneves@esenfc.pt (H.N.); 6 CINTESIS@RISE, Health Sciences Research Unit: School of Health, School of Health Sciences, University of Aveiro, 3810-198 Aveiro, Portugal; joaoptavares@ua.pt

**Keywords:** registered nurses, gerontogeriatric nursing, competency-based education, psychometrics, validation study

## Abstract

Background: As the aging population grows, facing multifaceted health challenges and escalating care costs, equipping newly graduated nurses with the requisite skills for high-quality gerontogeriatric care becomes crucial. This study assesses the psychometric properties of a Gerontogeriatric Competency (GGC) scale to evaluate the competencies of newly graduated registered nurses (RNs). Methods: Using a convenience sampling approach, a nationwide, observational prospective cohort study was conducted among 272 newly graduated RNs. The evaluation framework included a sociodemographic questionnaire, three groups of questions targeting gerontogeriatric nursing education aspects, and the GGC scale, with 64 competencies. Construct validity (via confirmatory factor analysis), known-group validity and reliability (assessed by Cronbach’s α) were examined. Results: The confirmatory factor analysis (CFA) showed an adequate index fit: the ratio of chi-square to degrees of freedom (χ^2^/df) = 2.785, the goodness-of-fit index (GFI) = 0.579, confirmatory fit index (CFI) = 0.864, the parsimony goodness-of-fit index (PGFI) = 0.526, the parsimony confirmatory-of-fit index (PCFI) = 0.809, the root mean square error of approximation (RMSEA) = 0.087, and the modified expected cross-validation index (MECVI) = 24.418. Differences were observed in gerontogeriatric competencies based on curriculum inclusion, self-confidence, knowledge in caring for older adults, and satisfaction with the nursing program content. The Cronbach’s α coefficient was 0.992 for the overall scale and ranged from 0.935 to 0.983 for the GGC dimensions. Conclusions: The GGC scale is a valid and reliable tool for assessing the gerontogeriatric competencies of new graduate RNs, highlighting its potential to enhance education, training, and, ultimately, the quality of care provided to the older population.

## 1. Introduction

The latest demographic projections from EUROSTAT [1] show an increasing aging population, especially in Europe, where it is estimated that between 2020 and 2030, there will be an increase of 16 million people aged 65 and over, and an additional increase of 15 million in the next decade. Portugal ranks as the fourth highest in the European Union in terms of the proportion of older adults [2]. This scenario poses challenges and opportunities in healthcare, social support, and economic sectors, emphasizing the need for comprehensive policies to manage the implications of an aging society effectively. In January of 2024, a Portuguese government plan was published, titled “Plano de Ação do Envelhecimento Ativo e Saudável 2023–2026” (Active and Healthy Aging Strategic Plan 2023–2026) [2], which addresses the demographic challenge of an aging population. It is structured around six key pillars to enhance quality of life, promote health, support social inclusion, ensure financial sustainability, and leverage the opportunities of an aging society. These pillars are health and well-being, autonomy and independent living, lifelong development and learning, healthy working life throughout the life cycle, income and the economics of aging, and participation in society. This plan also emphasizes the importance of measures to improve the training and capacitation of nursing staff, specifically focusing on initiatives such as organizing courses for professionals on the theme of active aging. These efforts are intended to improve the patients’ quality of care, the nurses’ working conditions and career prospects. This approach underlines the importance of gerontogeriatric competencies in ensuring that nurses are well-prepared to meet the unique needs of the older population, ultimately contributing to the broader goals of the action plan.

This aging phenomenon, associated with epidemiological transition (higher prevalence of chronic disease, geriatric syndromes, and multimorbidity), requires professionals with competencies in the care for older adults, especially, to respond to the complexity and specificity that care of this population needs. The nurses caring for older adults must be prepared with knowledge and skills to manage various ailments. This necessity underscores the vital role that nursing competence plays in enhancing the quality of care and influencing clinical outcomes. Fox’s [3] study reported that newly graduated nurses had positive attitudes toward older adults but hesitated to work with them, highlighting the need for improved gerontogeriatric education in nursing programs to boost their willingness and competence in elder care. Lee et al. [4] also suggested that positive experiences with older adults, confidence in older adult care, and gerontology education positively affect nursing students’ career interests in gerontogeriatric nursing. In Portugal, gerontogeriatric education in nursing indicates a growing awareness and integration of gerontogeriatric content in nursing curricula but also shows areas needing improvement [5]. Moreover, gerontogeriatric-related competencies were identified in only two nursing programs, and 13 schools reported needing assistance to strengthen the gerontogeriatric content in their curriculum. This situation underscores the importance of improving knowledge and skills in gerontogeriatric nursing and developing a standard of competencies to enhance education and practice.

The recognition of the importance of nurse competency assessment is well documented in the nursing literature [6]. Meretoja et al. [7] emphasize the role of experience in developing nursing competencies, underlining the need to evaluate the competencies of newly graduated nurses. Several studies point out the significance of establishing competency standards within the domain of gerontogeriatric nursing and collectively assert that such standards are pivotal for several reasons: they guarantee that nurses possess the necessary skills and knowledge tailored for the care for older patients [8], are crucial for delivering superior care, especially considering the vital role of clinical competence in driving effective patient outcomes [9], serve as a benchmark for assessing the readiness of new graduates for their professional roles [10], and facilitate the identification of areas needing improvement, thereby enabling the customization of educational programs aimed at enhancing the capabilities of newly qualified nurses [11,12]. Although there is growing interest and efforts in integrating these competencies globally, progress is uneven [13]. An international collaborative effort is necessary for developing shared gerontological competencies to enhance nursing care for older adults.

Nursing competencies directly impact the quality of nursing practice and clinical outcomes [14]. Enhancing nursing competencies is crucial for patient outcomes and care quality, leading to better patient satisfaction and professional advancement in nursing [13,14]. Previous deficiencies were noted in new nurses: communication, leadership, critical thinking, and stress management [15,16]. Bing-Jonsson et al. [15] reported that health promotion, disease prevention, treatment, palliative care, ethics and regulation, evaluation and action, covering basic needs, communication and documentation, responsibility and action, cooperation and attitudes towards older people are relevant categories of competence and, therefore, important to learn. The necessity for a more precise method to evaluate gerontogeriatric competencies [16] points to the significance of creating the GGC assessment tool, which aims to ensure older adults receive care from nurses that are adept at managing gerontogeriatric care, improving overall healthcare outcomes. Indeed, the healthcare system would benefit from having a more proficient workforce, addressing the needs of one of its largest patient populations. As the healthcare system continues to adapt to the needs of an aging population, newly graduated nurses must be equipped with the knowledge, skills, and attitudes required to provide comprehensive care to older adults. This adaptation includes clinical competencies and qualities such as appropriate attitudes as members of the society, as indicated in a study on the qualities required for newly graduated visiting nurses [17].

The emphasis on gerontogeriatric competencies reflects a broader understanding of nurses’ critical role in enhancing the quality of care for older adults. Having a valid and reliable GGC scale that shows the main areas of competence, adapted to the reality of aging in Portugal, nursing education, and the healthcare system, is crucial. Additionally, it may represent a first step towards the professional regulation of this area by The Order of Nurses. The ability of professional regulatory bodies to implement accreditation and credentialing systems in this field is one of the key facilitators of the development of gerontogeriatric nursing. Therefore, this study aimed to analyze the psychometric properties of the GGC assessment tool for recently graduated RNs.

## 2. Materials and Methods

This was an observational, prospective cohort, nationwide study. Survey data were collected between July 2021 and January 2022 using an online questionnaire in a sample of newly graduated RNs. This study is part of a larger Portuguese project, “Competências gerontogeriátricas de enfermeiros(as) recém-licenciados” (Gerontogeriatric competencies of newly graduated nurses). The Ethics Committee of the Health Sciences Research Unit: Nursing (UICISA: E), of the Nursing School of Coimbra (Order Nº 776/04-2021) approved the research protocol.

### 2.1. Participants and Procedures

This study was conducted in Portugal and included all newly graduated RNs in 2021/2022. The inclusion criteria for the participants were the following: (a) graduation from nursing school in the last six months and (b) registration in The Order of Nurses. Newly graduated RNs were chosen for this study to understand the influence of gerontogeriatric training and education during graduation on their self-perception and intention to work with older individuals. This study employed convenience sampling to recruit participants, chosen for its practicality and suitability within the scope of nationwide research, targeting a specific demographic of healthcare professionals.

The initial sample included 272 nurses who met the eligibility criteria. However, after excluding 30 individuals who did not complete the questionnaire, this study’s sample size was narrowed down to 242 participants.

### 2.2. Instruments

The online questionnaire comprised three parts. The first included the sociodemographic characterization of the participants (age, sex, region, type of residency and family, living and contact with grandparents, and experience in caring for older persons). The second included a set of three groups of questions related to gerontogeriatric nursing education: (1) the nursing curriculum addressed the gerontogeriatric competencies (yes or no), (2) self-confidence and knowledge in caring for older adults (yes or no), and (3) (un)satisfaction with the preparation and with the gerontogeriatric content in the nursing program (unsatisfied or satisfied). These questions were used in the known-group validation because previous studies reported their influence on bolstering positive attitudes towards older adults, influencing career choice, and having an important role in integrating gerontogeriatric competencies [18,19,20].

The GGC scale was developed in two phases. The first phase included a literature review to create the dimensions and items. The second is the modified Delphi technique with a group of 35 experts in gerontogeriatric nursing (education, practice, and research), according to the recommendation of Diamond et al. [21]. The end of this process resulted in 64 competencies, spread across nine domains: communication (six competencies), ethics and deontology (six competencies), care for older adults (21 competencies), safety and quality (nine competencies), family and/or family caregiver (eight competencies), interdisciplinarity (four competencies), health promotions and disease prevention (three competencies), management (three competencies), and professional development (four competencies). The agreement percentage was 80–90% in 20 competencies and ≥90% in 46 competencies [5]. The GGC used a Likert scale of five points (1 = “Not competent” to 5 = “Extremely competent”). The following formula was applied: the sum of the items minus the minimum score possible, divided by the maximum score possible, minus the minimum score possible (e.g., for the global score (200 − 64)/(320 − 64) = 0.53) to calculate the scores of the global GGC and all domains, on a scale of 0–1. The higher scores represented higher levels of competencies.

### 2.3. Data Collection Procedures

In the first data collection phase, The Order of Nurses of Portugal sent and approved a request for collaboration and disclosure. Afterward, The Order of Nurses sent a hyperlink for the survey, containing the questionnaire and the informed consent form, using the email list of nurses who met the inclusion criteria. A short introduction, along with the study description and the link was also published on The Order of Nurses news website throughout the data collection phase. If the participants did not respond to the online questionnaire, a monthly reminder was sent to increase the response rate.

### 2.4. Analysis

The sample characteristics were analyzed using descriptive statistics reporting *n* (%) for categorical data, and the mean and standard deviation were used for the continuous variables.

In assessing construct validity, confirmatory factor analysis was used. The following indices were taken into account to assess the goodness-of-fit of the model [22]: the ratio chi-square and degrees of freedom (χ^2^/df), the goodness-of-fit index (GFI), the confirmatory fit index (CFI), the parsimony goodness-of-fit index (PGFI), the parsimony confirmatory-of-fit index (PCFI), the root mean square error of approximation (RMSEA), and the modified expected cross-validation index (MECVI). The established cutoff criteria were as follows [23]: (1) χ^2^/df values of 2.0 or less indicated a good fit, and values between 3.0 and 5.0 an acceptable fit; (2) GFI and CFI values of 0.90 or higher; (3) PGFI and PCFI values should be between 0.50 and 0.60 for a good fit, or higher; (4) RMSEA values up to 0.05 signified a close fit, less than 0.08 a reasonable fit, and less than 0.10 a mediocre fit.

In the known-group validity, Student’s t-tests were applied to compare the differences in means between the questions related to the competencies in the nursing curriculum and the core gerontogeriatric competencies.

This study’s internal consistency was assessed using Cronbach’s α coefficient. Cronbach α values greater than 0.70 suggest good internal consistency. On the other hand, values exceeding 0.90 indicate a very high level of internal consistency, demonstrating that the items on the scale consistently measure the same underlying concept or construct.

Statistical analysis was performed using IBM SPSS^®^ version 27, and AMOS software (v. 27). A significance level of *p* < 0.05 was used for all comparisons.

## 3. Results

### 3.1. Sample Characterization

Most of the sample was female (83.9%) and single (90.1%). The mean age of the participants was 24.71 ± 4.94 years old. One hundred ninety-three (79.8%) attended the nursing course in public higher education institutions (Table 1). Concerning contact with older adults, 59.1% lived with older adults, 91.3% had regular contact, and 88.8% had previous experiences caring for older adults (not relatives).

### 3.2. Confirmatory Factor Analysis

In the initial analysis, several indices indicated that the model did not meet the preferred standards for optimal adjustment: χ^2^/df = 3.235, GFI = 0.514, CFI = 0.828, PGFI = 0.474, PCFI = 0.786, RMSEA = 0.097, and MECVI = 28.062. The GFI was notably low, the CFI was below the acceptable threshold, and the RMSEA was above the maximum preferred level. These findings revealed that the initial global model indicated the need for refinement. After re-evaluating the model parameters, a new CFA was conducted to assess the independence model. The independence model (Table 2) showed improvement, with χ^2^/df = 2.785, GFI = 0.579, CFI = 0.864, PGFI = 0.526, PCFI = 0.809, RMSEA = 0.087, and MECVI = 24.418. The independence model’s chi-square value (χ^2^) was 5257.911, with a *p*-value of <0.001, indicating a statistically significant discrepancy χ^2^/df ratio of 2.785. CFI improved to 0.864, near the desired threshold of 0.9, the RMSEA was 0.087, and theMECVI value was 24.418.

The nine dimensions of the GGC model revealed varying levels of fit. Dimensions such as “Communication”, “Ethics and deontology”, “Safety and quality”, “Family and/or family caregiver”, and “Interdisciplinarity” demonstrated good index fits, with χ^2^/df ratios ≤2.0. The “Care for older adults” and “Professional development” dimensions showed acceptable fit levels, with χ^2^/df < 5. Across all dimensions, the CFI and GFI values were ≥0.9, and the RMSEA values were <0.08 for several dimensions, indicating good adjustments, while other dimensions indicated lower index fits. Specifically, the “Care for older adults” dimension stood out due to its significantly high χ^2^ value of 744.896 and a *p*-value of <0.001, indicating a pronounced discrepancy from the expected results. Its χ^2^/df ratio of 4.161, RMSEA of 0.115, and *p*-value of <0.001 underscored a significant model misfit. The MECVI for this dimension was 3.566, markedly higher than that for other dimensions, highlighting its inefficiency in explaining the variance and covariance within the data. Conversely, the “Interdisciplinarity” dimension was characterized by an exceptionally low χ^2^ value of 0.078 and a *p*-value of 0.779, suggesting an almost perfect alignment between the observed and expected frequencies. Its RMSEA value of <0.001, with a *p*-value of 0.835 and a notably low MECVI of 0.078 demonstrated the model’s high efficiency and excellent fit. The “Health promotions and disease prevention” and “Management” categories both had χ^2^ values of <0.001, indicating an excellent fit. Still, their RMSEA values were unusually high (1.063 and 1.001, respectively), which typically suggests a poor fit.

The reliability results showed a Cronbach’s α for the total scale of 0.992. Each dimension also ranges from 0.935 to 0.983 (Table 3).

### 3.3. Known-Group Validity

The RNs who studied at nursing schools that address gerontogeriatric competencies in the curriculum showed a significantly higher score on GGC-global (0.43 vs. 0.37, *p* < 0.01) and on all dimensions (Table A1). The newly graduated RNs who reported self-confidence and knowledge in caring for older adults had a significantly higher score on GGC-global (0.43 vs. 0.31, *p* < 0.01) and on all dimensions (Table A1). Concerning the (un)satisfaction with the gerontogeriatric content in the nursing program, the newly graduated RNs that considered the level preparation as satisfactory showed a significantly higher score in the GGC-global (0.42 vs. 0.35, *p* = 0.017) and in the dimensions: “Communication” (0.41 vs. 0.38, *p* = 0.038), “Care for older adults” (0.40 vs. 0.32, *p* < 0.01), “Safety and quality” (0.43 vs. 0.37, *p* = 0.038), “Family and/or family caregiver” (0.42 vs. 0.36, *p* = 0.038), and “Health promotion and disease prevention” (0.42 vs. 0.33, *p* < 0.01). No statistically significant differences were found between (un)satisfaction with the nursing program and the dimensions “Ethics and deontology”, “Interdisciplinarity”, “Management”, and “Professional development” (*p* > 0.05, Table A1).

## 4. Discussion

In our validation study of the psychometric properties of gerontogeriatric nurse competencies among 242 newly graduated RNs, we applied a comprehensive approach to evaluate the scale’s validity and reliability. Following the methodology suggested by Boateng et al. [24], we focused on two primary forms of validity: construct validity and known-groups validity.

To assess construct validity, we conducted a CFA on the GGC model. This analysis was crucial for examining the factor structure we hypothesized based on the competency framework. The CFA helped us evaluate how well the data fit the theoretical model, with particular attention to indices beyond the chi-square statistic, considering its sensitivity to sample size. We assessed the model’s fit using several indices, including the CFI and the RMSEA. These indices were selected for their ability to provide a multifaceted evaluation of the model’s fit. By employing a range of indices, we aimed to ensure a thorough understanding of how well the theoretical model represents the observed data. This approach acknowledges the complexity of psychometric modeling and the importance of a comprehensive assessment to validate the psychometric properties of gerontogeriatric nurse competencies.

The evaluation of model fit indices from the initial global model represented a complex view of its effectiveness in capturing the data. The χ^2^/df = 3.235 was within acceptable limits [22], suggesting the model fits the data. However, this metric alone does not comprehensively assess model fit. The GFI at 0.514, the CFI at 0.828, and the MECVI at 28.062 all highlighted areas of concern. Specifically, the GFI is significantly below the commonly accepted threshold of 0.9, indicating a poor fit between the hypothesized model and the observed data. While closer to the benchmark of ≥0.9, the CFI still falls short, suggesting that the model’s ability to reproduce the observed data is not optimal. The high MECVI value further implies that the model may not hold well in cross-validation with independent samples, indicating concerns over its external validity and stability. The RMSEA at 0.097 exceeded the preferred maximum of 0.08 [22], which means a less-than-ideal fit and suggests that the model may not adequately account for the complexity of the data structure.

Thus, it was crucial to explore the underlying factors contributing to the inadequate model performance to address the concerns identified by the initial model fit indices. The low GFI suggests that the model did not sufficiently capture the covariances among the observed variables, likely due to the multidimensional nature of the assessed competencies, which do not cohere, as anticipated in the model’s structure. The suboptimal CFI indicated missing crucial pathways or interactions in our conceptual framework, which are essential for accurately replicating the observed data patterns. Furthermore, the elevated RMSEA underscored a significant misfit, potentially due to complex interrelations among competencies not effectively represented by the current model. The complexity of the model likely played a critical role, as it encompasses a broad spectrum of competencies, some of which could be inadequately defined or overlap with others, potentially confusing respondents and affecting response clarity. In addition, the specific demographic of our sample might have influenced these indices, given that their experiences and understandings of professional competencies could significantly differ from those of more seasoned practitioners.

In response to these initial findings, an independence model was developed. We undertook a review of the model to identify areas for improvement. This process involved analyzing the model’s structure without the constraints of the initial model, focusing solely on the relationships among variables, as dictated by theory, without any modifications suggested by the initial data. The independence model demonstrated improvements in fit indices. The χ^2^/df reduced to 2.785, indicating a better fit to the data. The GFI increased to 0.579, and while still below the ideal threshold, it marks an improvement from the initial model. The CFI improved to 0.864, moving closer to the ≥0.9 benchmark, which reflects a better model fit. The RMSEA decreased to 0.087, which, although still above the desired threshold, shows an improvement in model approximation error. The MECVI also declined to 24.418, suggesting improved external validity and stability for the independence model [22].

The complexity inherent in the GGC framework, characterized by its nine dimensions and a multitude of 64 competencies, poses a significant challenge in achieving a high-quality adjustment model. Such complexity could hinder the model’s ability to accurately mirror the underlying theoretical construct, a common concern in developing comprehensive frameworks [25,26]. Despite these challenges, applying CFA on a dimension-by-dimension basis revealed a more favorable outcome, demonstrating an adequate to good quality adjustment. This result indicates that the GGC framework is navigable, with different dimensions showing varying degrees of statistical fit [27,28]. 

A detailed analysis of the model fit indices for the GGC’s nine dimensions presents varied statistical robustness. Specifically, dimensions such as “Communication”, “Ethics and deontology”, “Safety and quality”, “Family and/or family caregiver”, and “Interdisciplinarity” showcased strong model fits, with χ^2^/df values of 2.0 or lower, indicating a solid alignment between the model and the observed data within these specific domains [22,28]. The “Care for older adults” and “Professional development” dimensions also displayed acceptable fit levels, with χ^2^/df ratios below 5, further affirming the framework’s statistical validity. Importantly, the CFI and GFI values met or exceeded the 0.9 benchmark across all dimensions, aligning with established criteria for a good model adjustment [29,30]. For the RMSEA, values below 0.08 for dimensions such as “Communication”, “Ethics and deontology”, “Safety and quality”, “Family and/or family caregiver”, “Interdisciplinarity”, and “Professional development” reflect a satisfactory model fit, conforming to the recommended standards for good fit [31]. Conversely, dimensions such as “Care for older adults”, “Health promotions and disease prevention”, and “Management” did not achieve this standard, indicating areas where the model fit could be improved. The GGC framework, through its detailed and differentiated statistical performance across various dimensions, validates its comprehensive coverage, while still offering precise insights for researchers.

This study’s second key finding is the GGC scale’s high internal consistency, evidenced by Cronbach’s α for the total scale, which was 0.992, indicating strong item coherence. This result suggests that the scale effectively measures a single, cohesive construct. The dimensions of the GGC scale also show strong reliability, with Cronbach’s α ranging from 0.935 to 0.983. Such consistent reliability across the scale and its dimensions indicates a well-constructed instrument, capable of producing reliable and trustworthy results. This high level of consistency ensures that the scale is a robust tool for researchers investigating GGC, allowing for dependable analysis of its relationships with other variables and applicability across various populations or contexts.

Alongside construct validity, we also explored known-groups validity, which assesses whether the scale can distinguish between groups that it should theoretically differentiate. In this context, we examined differences among newly graduated RNs based on their exposure to gerontogeriatric content during their training. Our findings revealed significant differences in competencies, confidence, knowledge, and satisfaction with the gerontogeriatric content among nurses who graduated from programs incorporating these competencies into their curriculum compared to those who did not. This result supports the scale’s known-groups validity, indicating that the GGC scale is sensitive to variations in gerontogeriatric education among new graduates.

The significant differences observed among newly graduated RNs showed that those who graduated from nursing programs incorporating gerontogeriatric competencies into their curriculum scored significantly higher on GGC-global than those who did not. This difference was observed across all dimensions of GGC, indicating that integrating gerontogeriatric education into nursing programs can substantially enhance graduates’ competencies in providing care to older adults. This finding aligns well with previous research, indicating a consistent pattern across studies, emphasizing the importance of a well-structured curriculum that focuses on gerontogeriatric competencies and targeted education in nursing [32,33]. While a general nursing curriculum provides a foundation, specialized courses in gerontogeriatric nursing ensure that students have an in-depth understanding of the unique aspects of older adult care [34].

The current scenario of nursing education shows a stark mismatch between theoretical learning and practical application, and the consequences of this educational imbalance include limited clinical exposure and challenges in applying theoretical knowledge in practice for nursing students, thereby affecting their capacity to deliver quality care in gerontogeriatric nursing [35,36,37]. Moreover, realizing the importance of learning about geriatric care often comes too late, after graduation, highlighting the critical need for enhancements in nursing education [19]. The gerontogeriatric competencies, encompassing communication, ethics and deontological practices, care for older adults, safety and quality measures, support for families and caregivers, interdisciplinarity, health promotion and disease prevention, management, and professional development, are integral to fostering a holistic approach to gerontogeriatric nursing [13]. These competencies not only enhance the care experience for older adults but also contribute to the professional growth of nurses, the efficiency of healthcare systems, and the overall improvement of public health outcomes for aging populations.

The validation of the GGC scale holds critical importance due to the increasing aging population and the urgent demand for nurses skilled in culturally, socially, and linguistically aligned competencies. This development supports the creation of better training programs, curricular enhancements, and evidence-based policy decisions. Furthermore, introducing the first Portuguese-validated gerontogeriatric nursing competency framework marks a critical step towards globalizing the standards of older adult care. It underscores the importance of developing and validating competency frameworks in various languages and cultural contexts to ensure that nursing education and practice across the globe are equipped to meet the diverse needs of aging populations. This study opens doors for comparative analyses and cross-cultural research, enriching the global discourse on gerontogeriatric nursing education and practice.

### Strengths and Limitations

This study is marked by several strengths, including its comprehensive evaluation of the psychometric properties of the GGC instrument, which ensures the tool’s reliability and validity for assessing newly graduated nurses’ competencies in gerontogeriatric care. With a significant sample size of 242 participants, this study enhances the generalizability of its findings within Portugal. Its nationwide approach makes the findings more representative of the population. The instrument displayed exceptionally high internal consistency, indicating that the items are highly correlated and effectively measure the same underlying concept. Additionally, this study addresses an urgent need for validated competency assessment tools in gerontogeriatric nursing education, given the aging population.

The dimensions “Care for older adults” and “Professional development” demonstrated lower fit levels within the GGC model. Particularly, the “Care for older adults” dimension highlighted a significant misfit, with a high χ^2^ value, χ^2^/df ratio, and RMSEA, indicating a pronounced discrepancy and inefficiency in explaining the variance. Conversely, “Communication” and “Interdisciplinarity” showed strong fits. The latter exhibited an almost perfect alignment, indicated by exceptionally low χ^2^ and RMSEA values, underscoring its high efficiency and excellent model fit. Exploratory factor analysis or additional pilot testing could refine the scale and enhance the model’s congruence with the empirical data.

Even with this, this study faces limitations, primarily due to its convenience sampling method, which may restrict the broader applicability of the findings due to potential sampling bias. To mitigate this possible bias, employing probability sampling techniques in future research could significantly enhance the generalizability of similar studies. The absence of a comparative analysis with other gerontogeriatric competency tools limits insights into the unique efficacy and positioning of the GGC instrument. This study’s cross-sectional design hinders the examination of competency development over time among newly graduated RNs. The reliance on online self-reporting introduces the possibility of response bias, with participants potentially overestimating their competencies. Additional studies, especially those employing at least five participants per competency item, and longitudinal evaluations in diverse contexts are essential to bolster the GGC’s validity and reliability and confirm its psychometric properties.

## 5. Conclusions

In this study, the GGC instrument was validated for newly graduated RNs. The psychometric properties show that the instrument is valid across its nine dimensions, using 64 items to assess the GGC competencies. Moreover, the instrument had excellent Cronbach’s α coefficients. This work significantly contributes to gerontogeriatric nursing education by offering a reliable tool for evaluating and shaping curricula and training programs. It addresses the critical demand for qualified nursing professionals capable of caring for the aging population, a priority especially relevant in Portugal, which may extend to other European countries.

## Figures and Tables

**Table 1:** Sociodemographic characterization.

	*n* ^1^ (%)
Sex	
Male	39 (16.1%)
Female	203 (83.9%)
Civil status	
Single	218 (90.1%)
Married/civil union	23 (9.5%)
Divorced/separated	1 (0.4%)
Nursing school type	
Private	49 (20.2%)
Public	193 (79.8%)
Geographical area where they usually live	
Alentejo	6 (2.5%)
Algarve	11 (4.5%)
Metropolitan Area of Lisbon	47 (19.4%)
Center	122 (50.4%)
North	50 (20.7%)
Autonomous Region of Madeira	3 (1.2%)
Autonomous Region of the Azores	3 (1.2%)
Lived with older adults	
Yes	99 (40.9%)
No	143 (59.1%)
Regular contact with older adults	
Yes	221 (91.3%)
No	21 (8.7%)
Previous experience caring for older adults (not relatives)	
Yes	215 (88.8%)
No	27 (11.2%)

^1^ *n*: sample size.

**Table 2:** Goodness-of-fit indexes for the nine-dimensional GGC model.

Number of Competencies	Dimensions	χ^2^; *p*-Value; χ^2^/df	CFI; PCFI	GFI; PGFI	RMSEA; *p*-Value	MECVI
6	Communication	9.971; 0.126; 1.662	0.997; 0.399	0.987; 0.282	0.052; 0.407	0.170
6	Ethics and deontology	9.920; 0.128; 1.653	0.998; 0.399	0.986; 0.282	0.053; 0.406	0.172
21	Care for older adults	744.896; <0.001; 4.161	0.919; 0.783	0.758; 0.588	0.115; <0.001	3.566
9	Safety and quality	32.143; 0.057; 1.531	0.995; 0.581	0.971; 0.453	0.047; 0.519	0.347
8	Family and/or family caregiver	18.231; 0.149; 1.402	0.998; 0.463	0.981; 0.354	0.041; 0.589	0.279
4	Interdisciplinarity	0.078; 0.779; 0.078	1.000; 0.167	1.000; 0.100	<0.001; 0.835	0.078
3	Health promotions and disease prevention	<0.001; --; --	1.000; <0.001	1.000; <0.001	1.063; <0.001	0.052
3	Management	<0.001; --; --	1.000; <0.001	1.000; <0.001	1.001; <0.001	0.052
4	Professional development	4.212; 0.122; 2.106	0.998; 0.333	0.992; 0.198	0.068; 0.273	0.087
	GGC-global	6198.796; <0.001; 3.235	0.828; 0.786	0.514; 0.474	0.097; <0.001	28.062
	GGC-independence model	5257.911; <0.001; 2.785	0.864; 0.809	0.579; 0.526	0.087; <0.001	24.418

**Table 3:** Internal consistency of the GGC (*n* = 242).

Number of Competencies	Dimensions	Mean	SD ^1^	Cronbach’s α
6	Communication	2.61	0.74	0.935
6	Ethics and deontology	2.95	0.77	0.965
21	Care for older adults	2.57	0.74	0.983
9	Safety and quality	2.69	0.74	0.957
8	Family and/or family caregiver	2.65	0.75	0.973
4	Interdisciplinarity	2.69	0.77	0.958
3	Health promotions and disease prevention	2.65	0.78	0.960
3	Management	2.50	0.85	0.948
4	Professional development	2.66	0.75	0.950
	GGC-global			0.992

^1^ SD: standard deviation.

## Data Availability

The data presented in this study are available in this article.

## Appendix A

**Table A1:** Statistical differences between gerontogeriatric competencies, self-confidence and knowledge, (un)satisfaction with gerontogeriatric content and the GGC-global.

Dimensions	Gerontogeriatric Competencies in the Curriculum	GGC M ± SD ^1^	Statistical Test	Self-Confidence and Knowledge to Care for Older Adults	GGC M ± SD ^1^	Statistical Test	(Un)Satisfaction with Gerontogeriatric Content in the Nursing Program	GGC M ± SD ^1^	Statistical Test
GGC-global	No	0.37 ± 0.16	t(240) = −2.73 *p* < 0.01	No	0.31 ± 0.18	t(54.3) = −4.24 *p* < 0.01	Unsatisfied	0.35 ± 0.13	t(45.8) = −2.48 *p* = 0.017
	Yes	0.43 ± 0.17		Yes	0.43 ± 0.17		Satisfied	0.42 ± 0.18	
Communication	No	0.35 ± 0.17	t(240) = −3.0 *p* < 0.01	No	0.29 ± 0.16	t(54.3) = −4.58 *p* < 0.01	Unsatisfied	0.34 ± 0.16	t(240) = −2.08 *p* = 0.038
	Yes	0.43 ± 0.19		Yes	0.42 ± 0.18		Satisfied	0.41 ± 0.19	
Ethics and deontology	No	0.47 ± 0.18	t(240) = −1.22 *p* = 0.209	No	0.41 ± 0.17	t(240) = −2.9 *p* < 0.01	Unsatisfied	0.46 ± 0.15	t(45.58) = −0.96 *p* = 0.337
	Yes	0.50 ± 0.20		Yes	0.50 ± 0.19		Satisfied	0.49 ± 0.20	
Care for older adults	No	0.35 ± 0.17	t(168.1) = −2.65 *p* < 0.01	No	0.29 ± 0.17	t(240) = −4.03 *p* < 0.01	Unsatisfied	0.32 ± 0.14	t(45.8) = −2.78 *p* < 0.01
	Yes	0.42 ± 0.19		Yes	0.41 ± 0.18		Satisfied	0.40 ± 0.19	
Safety and quality	No	0.38 ± 0.17	t(240) = −2.6 *p* = 0.01	No	0.33 ± 0.17	t(240) = −3.4 *p* < 0.01	Unsatisfied	0.37 ± 0.14	t(44.25) = −2.14 *p* = 0.038
	Yes	0.44 ± 0.19		Yes	0.44 ± 0.18		Satisfied	0.43 ± 0.19	
Family and/or family caregiver	No	0.37 ± 0.19	t(240) = −2.78 *p* = 0.011	No	0.30 ± 0.18	t(240) = −4.31 *p* < 0.01	Unsatisfied	0.36 ± 0.15	t(44.5) = −2.14 *p* = 0.038
	Yes	0.43 ± 0.18		Yes	0.43 ± 0.18		Satisfied	0.42 ± 0.19	
Interdisciplinarity	No	0.37 ± 0.19	t(240) = −2.73 *p* < 0.01	No	0.31 ± 0.17	t(54.7) = −4.38 *p* < 0.01	Unsatisfied	0.38 ± 0.15	t(240) = −1.31 *p* = 0.192
	Yes	0.45 ± 0.19		Yes	0.44 ± 0.19		Satisfied	0.43 ± 0.20	
Health promotions and disease prevention	No	0.37 ± 0.20	t(240) = −2.29 *p* = 0.023	No	0.30 ± 0.18	t(240) = −4.11 *p* < 0.01	Unsatisfied	0.33 ± 0.17	t(240) = −2.38 *p* < 0.01
	Yes	0.43 ± 0.19		Yes	0.43 ± 0.19		Satisfied	0.42 ± 0.20	
Management	No	0.32 ± 0.22	t(240) = −2.74 *p* < 0.01	No	0.25 ± 0.19	t(55.8) = −4.5 *p* < 0.01	Unsatisfied	0.33 ± 0.18	t(240) = −1.21 *p* = 0.111
	Yes	0.40 ± 0.20		Yes	0.40 ± 0.21		Satisfied	0.38 ± 0.22	
Professional development	No	0.37 ± 0.18	t(240) = −2.52 *p* = 0.012	No	0.31 ± 0.15	t(240) = −3.9 *p* < 0.01	Unsatisfied	0.37 ± 0.16	t(240) = −1.29 *p* = 0.2
	Yes	0.43 ± 0.19		Yes	0.43 ± 0.18		Satisfied	0.42 ± 0.19	

^1^ M ± SD: mean ± standard deviation.
